# Fracture mechanics modeling of aortic dissection

**DOI:** 10.1007/s10237-024-01845-3

**Published:** 2024-04-24

**Authors:** Ram Hemanth Yeerella, Shengqiang Cai

**Affiliations:** 1 Department of Mechanical and Aerospace Engineering, University of California San Diego, 9500 Gilman Dr, La Jolla, CA 92093 USA; 2 Program in Materials Science and Engineering, University of California San Diego, 9500 Gilman Dr, La Jolla, CA 92093 USA

**Keywords:** Aortic dissection, Tear propagation, Fracture mechanics, Energy release rate

## Abstract

Aortic dissection, a critical cardiovascular condition with life-threatening implications, is distinguished by the development of a tear and its propagation within the aortic wall. A thorough understanding of the initiation and progression of these tears, or cracks, is essential for accurate diagnosis and effective treatment. This paper undertakes a fracture mechanics approach to delve into the mechanics of tear propagation in aortic dissection. Our objective is to elucidate the impact of geometric and material parameters, providing valuable insights into the determinants of this pivotal cardiovascular event. Through our investigation, we have gained an understanding of how various parameters influence the energy release rate for tear propagation in both longitudinal and circumferential directions, aligning our findings with clinical data.

Introduction

Aortic dissection often begins with a tear or disruption in the aortic wall’s intima, the innermost layer. This tear allows blood to enter and flow between the layers of the aortic wall, forming a separate channel called the false lumen that runs alongside the normal aortic channel but is separated by the layers of the aortic wall called the intimal flap (Nienaber et al. 2016; Brunet et al. 2021; Rajagopal et al. 2007). The false lumen’s extent is contingent upon variables such as tear location, size, and hemodynamic factors. The intricate biomechanical complexities underlying the initiation and progression of aortic dissection demand a comprehensive understanding of the mechanical properties of the aorta, stress distributions, and the associated principles of fracture mechanics.

Mechanical modeling is indispensable in elucidating the nuanced biomechanical processes involved in aortic dissection. Diverse studies (Brunet et al. 2021; Rajagopal et al. 2007; Elger et al. 1996; Chuong and Fung 1986; Wang et al. 2023; Soleimani et al. 2023) are dedicated to unraveling these mechanics, enhancing the scope for preventive interventions. Finite element analysis (FEA) models (Raghavan and Vorp 2000; Gasser et al. 2005; Gasser and Holzapfel 2006; Volokh 2011; Di Achille et al. 2011; Ahamed et al. 2016; Azar et al. 2018), using isotropic hyperelastic solids or multilayer anisotropic hyperelastic solids, have been explored to analyze the stress distribution in aortic wall stresses and rupture propensities. Geometry is one of the predominant inputs for biomechanical simulations of the aortic system (Xu et al. 2020), and patient-specific models (Raut et al. 2013; Alimohammadi et al. 2014; Erhart et al. 2015; Shang et al. 2015; Subramaniam et al. 2020; Doyle et al. 2010), accounting for geometric variations, contribute significantly to discerning rupture locations and patterns of progression. While these models have provided valuable insights into assessing the risk of rupture, they primarily rely on stress analysis, which may not adequately predict the conditions necessary for crack propagation, a critical process of aortic dissection.

To comprehend the driving force behind tear propagation and improve the estimation of aortic dissection risk, fracture mechanics modeling is essential. In recent investigations, Wang et al. (2015) leveraged an energy-based approach to analyze the crack propagation in planar rectangular soft tissues, relating the energy release rate with crack length and applied stress. Building upon this research, Wang et al. (2016, 2017) further investigated aortic crack propagation in radial and circumferential directions, varying the widths and depths of the tear using XFEM in ABAQUS. Their findings indicated that shallow and elongated cracks tend to buckle, with potential for crack arrest, whereas deeper cracks are more inclined to propagate radially. In reality, the media is comprised of many elastic lamellar units, and radial crack propagation across the entire lamellae is not energetically favored (Gültekin et al. 2019).

In this study, we adopt fracture mechanics theory to quantify the conditions for crack propagation along longitudinal and circumferential directions in aortic dissection. We compare the driving force for crack propagation, represented by the energy release rate (*G*), with the resistance, characterized by the fracture energy of the aorta ($\Gamma$). As long as the calculated driving force is lower than the resistance ($\Gamma$), crack propagation would be inhibited (Griffith 1921; Irwin and Wells 1965). Formulating a comprehensive model that incorporates all material properties alongside real-life geometrical considerations presents a significant challenge. Our approach, therefore, takes a simplified yet insightful route. We embark on a journey through the fundamental principles of fracture mechanics, specifically applied to type B aortic dissection (Stanford classification), where the false lumen runs parallel to the true lumen in the descending aorta. Our investigation specifically explores how geometrical parameters and material properties influence crack propagation, calculating *G* semi-analytically along both the longitudinal and circumferential directions. Our model’s results align favorably with clinical observations, indicating that a lower wall thickness-to-radius ratio and thinner false lumen result in a heightened energy release rate for tear propagation. Furthermore, our findings suggest that *G* in the longitudinal direction initially decreases and later increases as the tear widens, while it monotonically increases for circumferential spread of the tear. Based on these results, we generated a safety plot to predict the risk of aortic dissection with tear geometry at different blood pressure levels.

## Fracture mechanics model

**Fig. 1:**
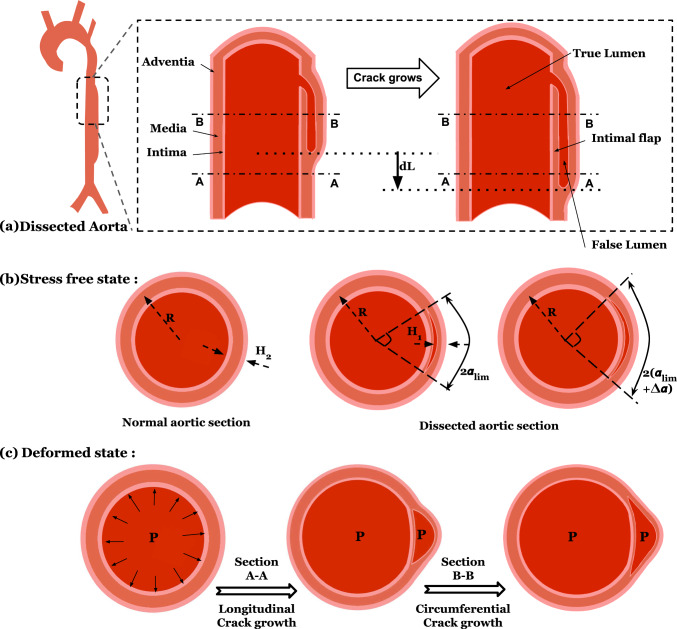
Aortic dissection depiction. **a** Crack propagation in the longitudinal direction **b** Stress-free (undeformed) state of normal section and dissected section of the aorta for two tears of different sizes. *R*, $H_1$, and $H_2$ correspond to the radius, false lumen thickness, and true lumen thickness of the aortic wall, respectively, and $\alpha _{\text {lim}}$ and $\alpha _{\text {lim}} + \Delta \alpha$ are the crack angles of the two tears. **c** Deformed state of normal section and dissected section of the aorta. During longitudinal tear propagation, section A–A changes from a normal section to a dissected section, as shown. When the tear propagates circumferentially, increasing the crack length, section B–B changes as depicted

Assuming a plane strain deformation in the aorta, the fracture analysis simplifies to a 2D model as shown in Fig. 1. Consider a section of the aorta devoid of any crack; the aorta is modeled as an annular ring with a true lumen wall thickness TLWT) ‘$H_2$,’ which is subjected to a physiological blood pressure ‘P’ on the inner wall of the true lumen. *R* is the mean radius of the annular ring where the inner wall surface has a radius of *R* – $H_2$/2, and the outer wall radius is *R* + $H_2$/2. For the dissected section of the aorta, the crack region is described by an angle of 2$\alpha _{\text {lim}}$ and a false lumen wall thickness (FLWT) of $H_1$. Here, $\alpha _{\text {lim}}$ is defined as the crack angle of the tear.

### Energy release rate

The energy release rate is defined as the reduction of total potential energy per unit increase in crack area. The deformation is two-dimensional in our model with the assumption of plane strain. Thus, we calculate the energy release rate (*G*) as the ratio of change in the total potential energy per unit length (U) to the increase in crack length from the initial state to the final state (Anderson 2017; Kumar 2009; Gross and Seelig 2017; Taylor 2018. The total potential energy per unit length (*U*) is calculated as:

1 $$\begin{aligned} \text {U} = W_{\text {SE}} - P\Delta A_{\text {in}} \end{aligned}$$

here, $W_{\text {SE}} = \int _{A}^{} W \, dA$, integrating over the cross-section area of the aortic wall, and $\Delta A_{\text {in}}$ corresponds to the cross-section area change of the lumen (area enclosed by the inner aortic wall) before and after deformation. When a tear propagates, we calculate the aortic section’s potential energy before and after the steady tear propagation, as shown in Eq. (1).

#### Energy release rate for longitudinal tear propagation

When tear propagation is in the longitudinal direction, the initial state ${U}_{I}$ corresponds to the total potential energy of the normal section of the aorta per unit length, while ${U}_{II}$ corresponds to the dissected section of the aorta with a crack angle of $\alpha _{\text {lim}}$. In the stress-free state, the distance from the radial center to the crack surface is computed as the radius of the outer wall of the aorta—false lumen wall thickness ($H_1$), which is $(R+ H_2/2 - H_1)$. During the crack propagation, the total potential energy is reduced by converting the normal aorta to dissected one of the same lengths in the axial direction (dL) (Fig. 1a). The total crack length is taken as the arc length of the crack, i.e., $(R+ H_2/2 - H_1)*2\alpha _{\text {lim}}$, which is also the increase in crack length for longitudinal crack propagation. It is noted that the crack length is defined in the undeformed or stress-free state of the aorta. Therefore, the energy release rate for longitudinal tear propagation is expressed as follows:

2 $$\begin{aligned} G = \frac{\text {U}_{I} - \text {U}_{II}}{(R+ H_2/2 - H_1)*2\alpha _{\text {lim}}} \end{aligned}$$

#### Energy release rate for circumferential tear propagation

For circumferential tear propagation, an upwind scheme is employed to calculate the energy release rate. As the tear grows slightly in the circumferential direction, increasing the crack angle from $\alpha _{\text {lim}}$ to $\alpha _{\text {lim}} + \Delta \alpha$ (Fig. 1c), the total potential energy per unit length decreases from $U_{II}(\alpha _{\text {lim}}) \rightarrow U_{II}(\alpha _{\text {lim}} + \Delta \alpha )$, and the crack length increases by $(R+ H_2/2 - H_1)*2\Delta \alpha$. The G in the circumferential direction is computed as follows:

3 $$\begin{aligned} G = \frac{U_{II}(\alpha _{\text {lim}}) - U_{II}(\alpha _{\text {lim}} + \Delta \alpha )}{(R+ H_2/2 - H_1)*2\Delta \alpha } \end{aligned}$$

We set $\Delta \alpha$ to $4^{\circ }$ in our calculations.

### Material model

In this work, we assume the aorta to be an isotropic incompressible hyperelastic material in plane strain deformation, i.e., $\lambda _3 = \lambda _z = 1$. We first use the Neo-Hookean hyperelastic material model that assumes a perfect elasticity, whose strain energy density (Ogden 1984) is given by:

4 $$\begin{aligned} \begin{aligned}&W =\frac{\mu }{2}\left( \lambda _1^2 + \lambda _{2}^{2} - 2 \right) \end{aligned} \end{aligned}$$

The results obtained from the analytical model are compared with the FEA results to validate our approach. Blood vessels are not infinitely extensible, and the aortic wall is found to be stiffer with high blood pressure and at larger strains (Avolio 2013). To understand this, we also use the Gent hyperelastic model, which considers the strain stiffening owing to the chain inextensibility in the material, and the strain energy density (Gent 1996) is given by:

5 $$\begin{aligned} \begin{aligned}&W =- \frac{\mu J_m }{2} \ln \left( 1 - \frac{\lambda _1^2 + \lambda _{2}^{2} - 2 }{J_m} \right) \end{aligned} \end{aligned}$$

The parameter $J_m$ corresponds to the strain limitation. When $J_m$ is infinite, the Gent material model approaches the Neo-Hookean model.

Realistically, the aorta is not isotropic and is made of collagen fibers embedded in the elastin matrix, leading to the aortic wall’s anisotropy. The Holzapfel–Ogden–Gasser (HGO) model is proposed by Holzapfel et al. (2000) to describe arterial tissue properties better. The strain energy density is given as a summation of the isotropic strain energy density of the elastin, and the anisotropic part comes from the energy density of collagen fibers, which follow an exponential function of fiber stretch. Here, we use the following strain energy density function based on the standard HGO model given as (Karlsson et al. 2023):

6 $$\begin{aligned} \begin{aligned} W =&W_{\text {iso}} + W_{\text {aniso}}\\ =&\frac{\mu }{2}\left( \lambda _1^2 + \lambda _{2}^{2} + \lambda _{3}^{2} - 3 \right) + \frac{K_1}{K_2}\left( e^{K_2(I-1)^2} - 1 \right) \end{aligned} \end{aligned}$$

Here, $K_1$ is a stress-like parameter related to the collagen fiber stiffness, and $K_2$ is a dimensionless parameter related to strain stiffening of collagen fibers. In our model, the stretches in the aortic wall are taken as $\lambda _{1}$ = $\lambda _{\theta }$, $\lambda _{2}$ = $\lambda _{r}$ , and $\lambda _{3}$ = $\lambda _{z} = 1$. *I* is given as $\lambda _{\theta }^2\text {cos}^2(\beta ) + \lambda _{z}^2\text {sin}^2(\beta )$, where $\beta$ is the angle between the circumferential direction and the principal (mean) fiber direction in the unloaded configuration, which is $\approx 40-45^\circ$ (Astrand et al. 2011; Karlsson et al. 2023). Since we have $\lambda _z = 1$ and assuming $\beta = 45^\circ$, I simplifies as $\lambda _{\theta }^2/2 + 1/2$

### Analytical model and its validation

**Fig. 2:**
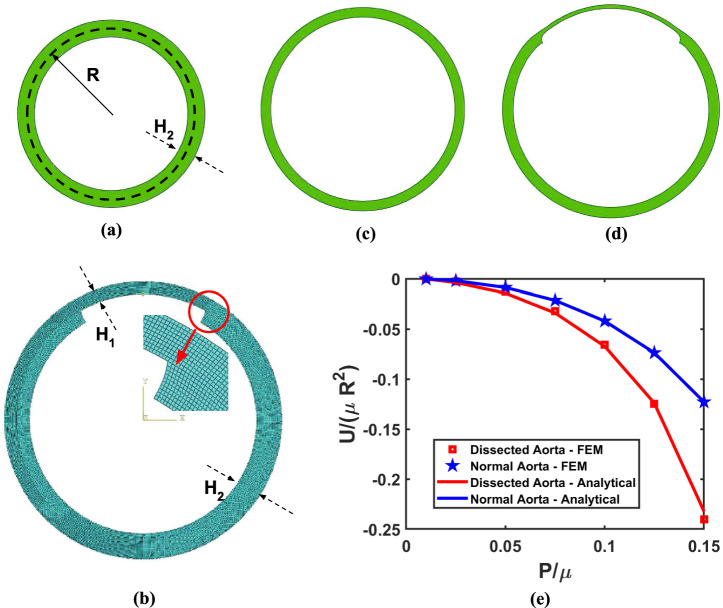
FEA Model **a** Undeformed section of the Normal aorta. **b** Meshed undeformed Part of the Dissected section of the aorta. **c** Deformed section of the Normal aorta. **d** Deformed section of the Dissected aorta. **e** Comparison of Total potential energy predicted by the analytical model with FEA results for Normal aortic section with $H_2$/R = 0.2 and Dissected aortic section with Crack angle ($\alpha _{\text {lim}}$) of $30^\circ$, $H_2$/R = 0.2 and $H_1$/$H_2$ = 0.5

For the normal aortic section without any crack due to axisymmetry, the problem simplifies to a 1D analysis along the radial direction, whose exact deformation and stress fields can be obtained (refer to Appendix A.1). In the presence of a crack, the section of the dissected aorta loses its axisymmetry. As pressurized blood flows through the tear and enters the false lumen, we assume that the inner wall of the false lumen experiences the same pressure as the true lumen. The contribution of the intimal flap between the true and false lumen to the total potential energy is neglected since it is subjected to uniform pressure ‘P’ on both sides. Disregarding the intimal flap, the region with a crack is modeled as an annulus sector (see Fig. 2b) with an angle of 2$\alpha _{\text {lim}}$ and a thickness of $H_1$. Here, $\alpha _{\text {lim}}$ is defined as the crack angle, and $H_1$ represents the false lumen wall thickness (FLWT). Treating this dissected aorta as a thin-wall membrane, we solve the planar equilibrium equations to obtain the deformation and stress field (refer to Appendix A.2).

To verify the appropriateness of our assumptions and the correctness of the coding, we initially validated our model by comparing the total potential energy of the normal and dissected aorta with the finite element analysis (FEA) results obtained from ABAQUS simulations, utilizing the Neo-Hookean material model. We utilized the CPE4RH element from the standard element library, which is a quad-shaped element belonging to the plane strain family with linear geometric order, hybrid formulation, reduced integration, and default settings for element controls. For discretizing the aorta part, we employed a fine mesh with an element size of approximately 0.01R, utilizing the free meshing technique and the medial axis algorithm, as depicted in Fig. 2b. A constant pressure $P/\mu$ was applied on the inner surface of the part. We selected a static general step considering nonlinear geometry effects with all default solver settings.

The deformed shapes of the normal and dissected section of the aorta are shown in Fig. 2c and d for $P/\mu = 0.1$. Figure 2e shows the total potential energy vs. pressure plot for normal aorta and dissected aorta. We observe a decrease in total potential energy with an increase in pressure for both the normal section and the dissected section of the aorta. As a result, the reduction in total potential energy increases with pressure. When the pressure increases, both the total strain energy and the potential energy of the blood pressure increase, with the latter being increasingly dominant at greater pressures. The results from our model and ABAQUS simulations have a minimal discrepancy. The small error in the predicted $U_{II}$ may be attributed to the membrane assumption in our analytical model. However, given that our model aligns well with the FEA results, we proceed to investigate the energy release rate for tear propagation along longitudinal and circumferential directions, along with the various parameters influencing it.

## Results

In this section, we first show the results of the variation of energy release rate for the tear propagation in the longitudinal and circumferential directions, treating the aorta as an isotropic material. Later, we discuss how the anisotropy in the aorta affects the energy release rate in both directions.

### Longitudinal tear propagation

**Fig. 3:**
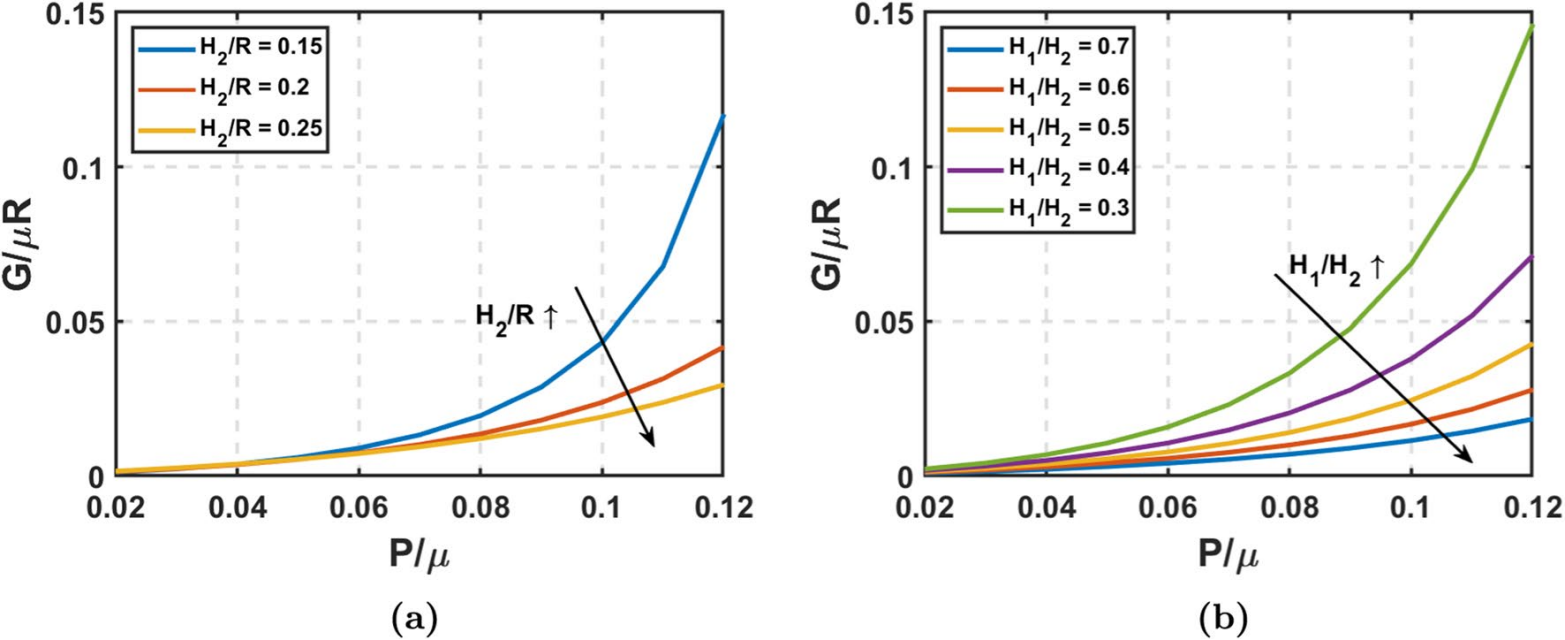
Longitudinal tear propagation: Neo-Hookean material: **a** Energy release rate versus Pressure with varying thickness ($H_2/R$) at crack angle ($\alpha _{\text {lim}}$) = $30^\circ$ and tear depth ($H_1$/$H_2$) = 0.5. **b** Energy release rate versus Pressure with varying tear depth($H_1/H_2$) at $\alpha _{\text {lim}}$ = $30^\circ$ and $H_2$/R = 0.2

Using a Neo-Hookean material model, we first examine the impact of aorta thickness and tear depth on energy release rate (*G*). Figures 3a and 3b illustrate how G along longitudinal direction changes with pressure when varying TLWT and FLWT, respectively. From Fig. 3, it is evident that the energy release rate (*G*) increases with pressure for tear propagation in the longitudinal direction, and the slope of this curve also increases with pressure. In Fig. 2e, we saw that the reduction in the total potential energy from normal to dissected aorta increases with pressure, thus increasing the longitudinal energy release rate with pressure, as the crack length remains constant. Figure 3a reveals that G declines as the aorta’s TLWT-to-radius ratio rises. Greater wall thickness reduces the stretching needed for the aortic wall to balance blood pressure. So, greater TLWT diminishes the magnitude of the total potential energy for both the normal section and the dissected section of the aorta, with the decrease being more pronounced in the latter. As TLWT increases, *G* consequently decreases, and this observation holds significance to the aging process. Studies (Jadidi et al. 2020) indicate that the aortic wall’s thickness-to-radius ratio decreases as individuals age, elevating the susceptibility to aortic dissections, which heightened G reflects with the reduction in $H_2/R$. Figure 3b elucidates the effect of FLWT on *G*. Deeper cracks yield an effectively thinner wall in the false lumen, reducing $H_1/H_2$. As previously discussed, the reduction in thickness increases the stretching of aortic walls to maintain pressure equilibrium. Deeper cracks indicate that the thickness reduces only in the false lumen, resulting in a more significant stretch for equilibrium. Consequently, the total potential energy of the normal aorta remains unchanged, while the total potential energy of the dissected aorta decreases significantly. This intensifies the reduction in total potential energy difference, leading to increased *G*.

**Fig. 4:**
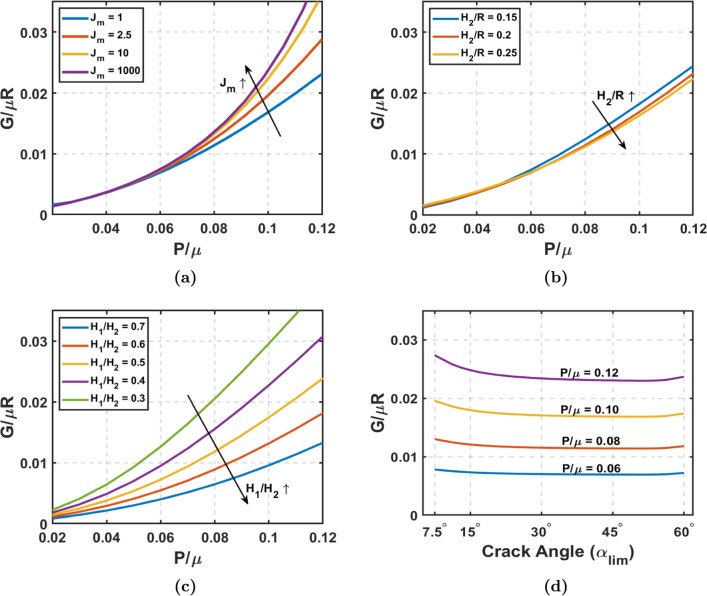
Longitudinal tear propagation with strain stiffening: Gent hyperelastic material: **a** Energy release rate versus Pressure with varying $J_{m}$ at $\alpha _{\text {lim}}$ = $30^\circ$, $H_2$/R = 0.2, and $H_1$/$H_2$ = 0.5. **b** Energy release rate versus Pressure with varying thickness ($H_2/R$) at $J_m = 1$, $\alpha _{\text {lim}}$ = $30^\circ$ , and $H_1$/$H_2$ = 0.5. **c** Energy release rate versus Pressure with varying tear depth($H_1/H_2$) at $J_m = 1$, $\alpha _{\text {lim}}$ = $30^\circ$ , and $H_2$/R = 0.2. **d** Energy release rate versus Crack angle ($\alpha _{\text {lim}}$) with varying pressure at $J_m = 1$, $H_2$/R = 0.2, and $H_1$/$H_2$ = 0.5

Considering the strain stiffening in soft tissues, we see how various factors affect *G* when using two parameters–Gent’s material model in Fig. 4. Figure 4a shows how the strain-stiffening parameter $J_m$ affects the G along the longitudinal direction. With a decrease in the maximum allowed strain $J_m$, *G* decreases in magnitude. *G* predicted from a Neo-Hookean material model is significantly higher than the one indicated with Gent’s model, which has a strain-stiffening effect. As we increase the $J_m$ to a very high number, the *G* indicated by the Gent model reaches the value predicted by the Neo-Hookean material model. The material has significant strain energy at relatively lower stretch values with a higher strain-stiffening effect (i.e., lower $J_m$ ). Consider a specific pressure value; the stretch needed to achieve equilibrium at this pressure will be lower when $J_m$ is lower. This leads to a reduced deformed area, decreasing the potential energy of pressure, but the strain energy will not decrease by the same extent. This reduces the magnitude of total potential energy, reducing *G* for a lower $J_m$. Figures 4b and 4c correspond to the plots of *G* versus pressure by varying the TLWT and FLWT, respectively, with $J_m$ = 1. In Gent’s model, when $J_m$ = 1, the variation of G with TLWT-to-radius ratio is relatively minor, which has notably affected the Neo-Hookean model. This impact is due to strain stiffening, which causes the membrane to experience reduced stretching in both normal and dissected aorta. As a result, the difference between $U_{I}$ and $U_{II}$ diminishes, which does not vary significantly with $H_2/R$. While we still notice a trend akin to the Neo-Hookean material scenario, the influence of the TLWT-to-radius ratio has been substantially attenuated. The reduction in *G* value given by the Neo-Hookean model to Gent’s model is more pronounced when the TLWT-to-radius ratio is lower. Conversely, the effect of tear depth remains significant, albeit with reduced magnitude, compared to the Neo-Hookean case. Referring to our discussion for Fig. 3b, when the $H_1/H_2$ is lower, the total potential energy for dissected aorta is significantly lower than that of the normal aorta, resulting in a higher *G*. Due to the material’s strain-stiffening characteristic, the stretch reduction is more prominent, thus reducing the change of the total potential energy in the presence of a crack as $H_1/H_2$ decreases. Although the trend still demonstrates a notably higher G for thinner false lumen, this impact is not as pronounced as in the Neo-Hookean material case. Figure 4d describes the variation of G the crack angle ($\alpha _{\text {lim}}$) at different pressure levels. Initially, when the crack angle is minimal, *G* is at its highest. As $\alpha _{\text {lim}}$ increases, *G* gradually decreases until it reaches a nearly constant value. Subsequently, when the crack angle further increases, *G* rises again. At a given pressure, the variation in *G* with crack angle primarily depends on the total potential energy of the dissected aortic section and the crack length because the total potential energy of the normal aortic section remains constant. As the crack angle increases, both the total strain energy and the deformed area increase, leading to a decrease in the total potential energy of the dissected aortic section. With the widening of the crack at increased angles, there is a notable increase in the disparity between the total potential energy of the dissected and normal aortic sections. Because both the difference in total potential energy and crack length increase, albeit at different rates, the plot of *G* versus crack angle exhibits a non-monotonic pattern.

### Circumferential tear propagation

**Fig. 5:**
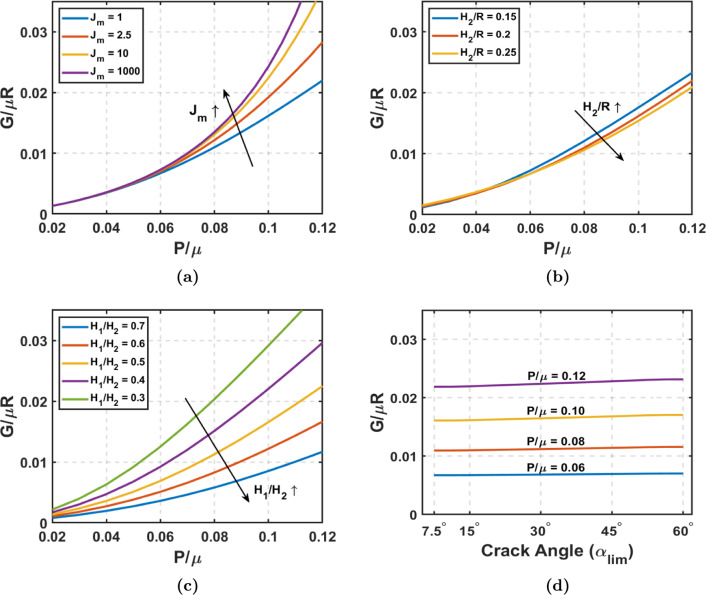
Circumferential tear propagation with strain stiffening: Gent hyperelastic material: **a** Energy release rate versus Pressure with varying $J_{m}$ at $\alpha _{\text {lim}}$ = $30^\circ$, $H_2$/R = 0.2, and $H_1$/$H_2$ = 0.5. **b** Energy release rate versus Pressure with varying thickness($H_2/R$) at $J_m = 1$, $\alpha _{\text {lim}}$ = $30^\circ$ , and $H_1$/$H_2$ = 0.5. **c** Energy release rate versus Pressure with varying tear depth($H_1/H_2$) at $J_m = 1$, $\alpha _{\text {lim}}$ = $30^\circ$ , and $H_2$/R = 0.2. **d** Energy release rate versus crack angle ($\alpha _{\text {lim}}$) with varying pressure at $J_m = 1$, $H_2$/R = 0.2, and $H_1$/$H_2$ = 0.5

Figure 5 shows the impact of various parameters on the G along the circumferential direction considering the strain-stiffening effect (the results of *G* in the circumferential direction for Neo-Hookean material are given in Fig. 9). In Fig. 5a, we observe the effect of strain-stiffening parameter $J_m$ on *G* along the circumferential direction. Figures 5b and 5c correspond to the plots of *G* versus pressure by varying the TLWT and FLWT, respectively, with $J_m$ = 1. Figures 5a, 5b, and 5c are plotted for crack angle = $30^\circ$ (initial state). Similar to the results in longitudinal tear propagation, *G* for circumferential tear propagation increases with $J_m$ and decreases with increasing $H_2/R$ and $H_1/H_2$. In Figure 5a, we see that for very high $J_m$, i.e., approaching the Neo-Hookean model (Fig. 9), the *G* predicted for the circumferential direction is higher than for the longitudinal direction counterpart. However, *G* is slightly lesser for stiffer material with low $J_m$ in the circumferential direction than along the longitudinal direction. Figures 5b and 5c look very similar to their counterparts from longitudinal crack propagation (Figs. 4b and 4c), with the only difference being slightly lower in magnitude. With increasing $H_2/R$, the difference in total potential energy due to circumferential tear propagation decreases due to reduced stretching, although this change is minimal. When the $H_1/H_2$ is lower, it leads to greater stretching in the false lumen wall as the crack grows circumferentially, having a greater difference between $U_{I}$ and $U_{II}$. As the $H_1/H_2$ ratio increases, the total potential energy difference decreases, thus reducing the *G*. Figure 5d shows the *G* versus crack angle plot at different pressures. The energy release rate monotonically increases with the crack angle ($\alpha _{\text {lim}}$) and has a steeper slope at higher pressures. As discussed earlier, with the increase in crack angle, the magnitude of total potential energy increases, and so does its difference for adjacent crack angles, but the increase in crack length is fixed, resulting in the monotonic rise of *G* as the crack widens along the circumferential direction.

### Effect of anisotropy

**Fig. 6:**
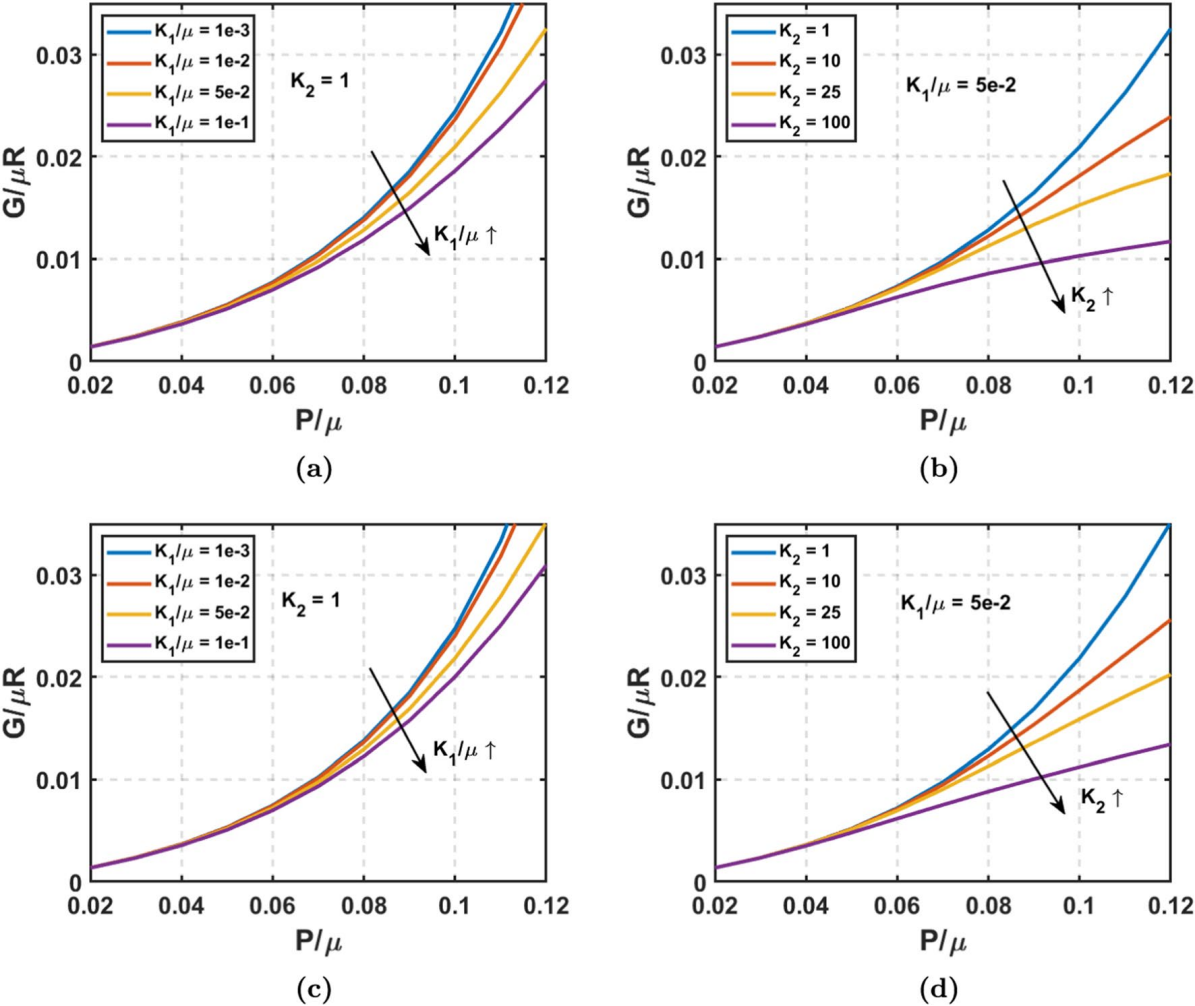
Energy release rate versus Pressure plots using anisotropic HGO model at $\alpha _{\text {lim}}$ = $30^\circ$, $H_2$/R = 0.2, and $H_1$/$H_2$ = 0.5 for longitudinal crack propagation: **a** Varying $K_1/\mu$ when $K_2$ = 1. **b** Varying $K_2$ when $K_1/\mu$ = 5e – 2. Energy release rate versus Pressure plots at $\alpha _{\text {lim}}$ = $30^\circ$, $H_2$/R = 0.2, and $H_1$/$H_2$ = 0.5 for circumferential crack propagation. **c** Varying $K_1/\mu$ when $K_2$ = 1. **d** Varying $K_2$ when $K_1/\mu$ = 5e – 2

Using the HGO material model, we investigated the effect of anisotropy and fiber stiffness by varying $K_1$ and $K_2$ parameters, and their corresponding energy release rate vs. pressure plots are shown in Fig. 6. Figure 6a and b corresponds to the variation of energy release rate with independently changing $K_1/\mu$ and $K_2$, respectively, for the longitudinal propagation of tear. Figure 6c and d shows the *G* versus pressure plots with the variation of $K_1/\mu$ and $K_2$ when the tear grows circumferentially. Through numerical simulations and experimental data, Huh et al. (2019) reported significant variations in the values of $\mu$, $K_1$, and $K_2$ across different age groups. In our study, we opted for a representative value of $K_2$ that is the same order of magnitude as the reported values when varying $K_1/\mu$ and vice versa. We observe that when the exponential coefficient value of $K_2$ is low, the energy release rate predicted by the HGO model approaches that of the Neo-Hookean model, both in longitudinal and circumferential directions, for a lower initial fiber modulus ($K_1$) value. We observe that as $K_1/\mu$ increases, *G* decreases, and this reduction with an increase in initial fiber modulus is greater for the longitudinal tear propagation than the circumferential tear propagation. Looking at the effect of the exponential coefficient $K_2$, we see that the energy release rate decreases with the increase in the $K_2$ value for a considerable value of $K_1/\mu$. As the $K_2$ increases, the stiffness of the fibers increases drastically at higher pressures, reducing the deformation and strain energy, resulting in lower total potential energy, thereby decreasing the energy release rate. This effect is similar to that of strain-stiffening parameter $J_m$ in the Gent model. Both these parameters increase the stiffness at higher material stretch and limit the strains. The effect of tear depth and true lumen thickness is akin to that described by Gent’s model, wherein reducing thickness or increasing tear depth amplifies the energy release rate at chosen $K_1/\mu$ and $K_2$ values. However, the impact of the former is considerably less pronounced than the latter’s. The results of these trends are shown in Fig. 10

## Discussion

The primary challenge in modeling aortic dissection fractures lies in individuals’ variability of material properties, geometry, and crack types. Statistical inferences by Paruchuri et al. (2015) and Keisler and Carter (2015) support the notion that the risk of abdominal aortic dissection increases when the aorta’s diameter exceeds 4.5–5 cm, with a significant risk reduction observed for diameters below 3.5 cm. Although there exists a correlation between aortic dissection risk and aortic diameter, it is important to note that this relationship is not entirely independent due to variations in the aortic diameter threshold across different age groups (Grimshaw and Thompson 1997; Boudoulas et al. 2018; Tadic et al. 2022). Additionally, the risk of aortic dissection inversely correlates with false lumen wall thickness (Shiran et al. 2012; Van Puyvelde et al. 2015). While the mean diameter and thickness of the aorta generally increase with age, the aorta stiffens with aging (Liu et al. 2015; Komutrattananont et al. 2019), necessitating consideration of all these changes in predicting aortic dissection.

In our study, we employed a semi-analytical approach to investigate how various factors influence the spread of aortic dissection. Our findings indicate that when we model the aorta as an isotropic and anisotropic solid, the energy release rate (*G*) increases with blood pressure, tear depth, and a decrease in the thickness of the lumen wall relative to the aorta radius. We observed that, under similar geometrical conditions, the predicted G from our model decreases as the aorta becomes stiffer. This finding is consistent with clinical observations of Grimshaw and Thompson (1997), suggesting a higher risk of aortic dissection for a specific diameter in a 60-year-old age group compared to a 75-year-old age group due to increased aortic stiffness. Furthermore, when modeling the aorta as a Gent hyperelastic material, as the crack angle increases, the energy release rate decreases along the longitudinal direction but increases along the circumferential direction.

**Fig. 7:**
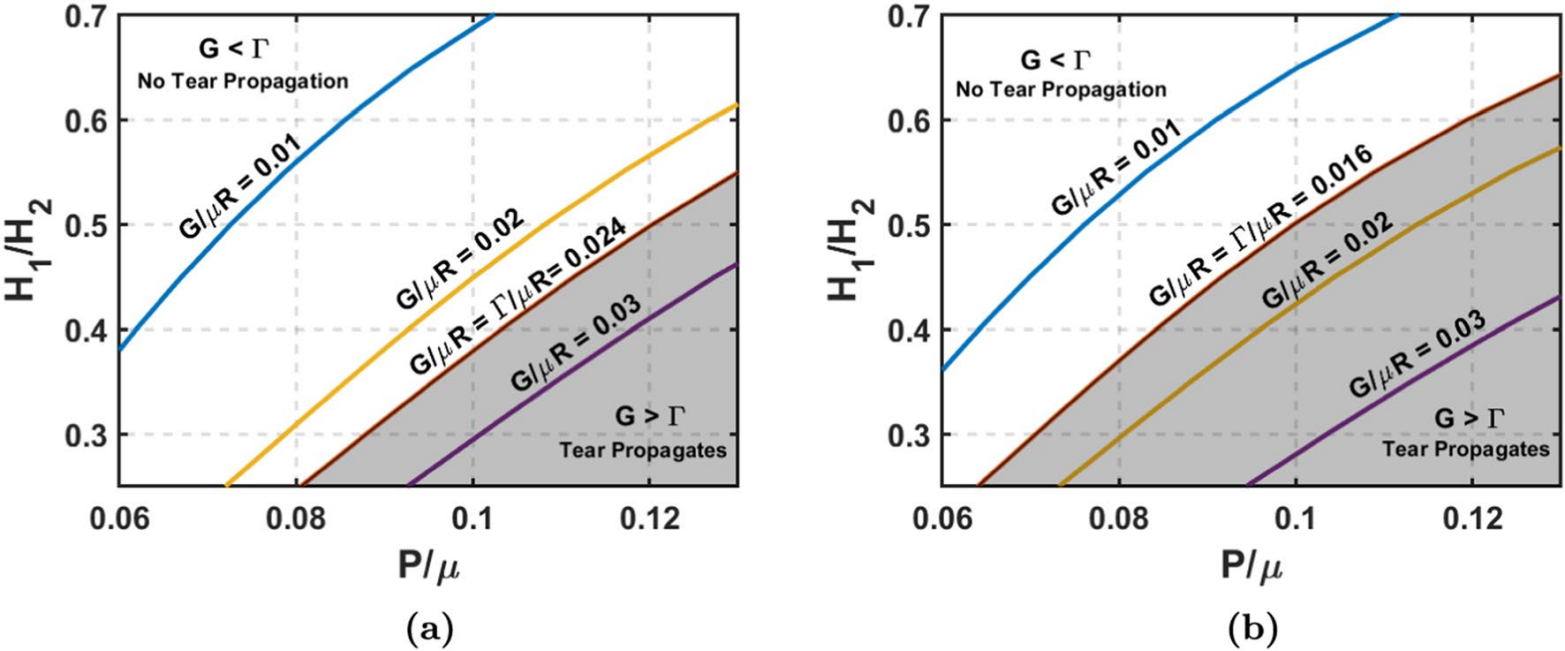
Safety plot for aortic dissection, with the gray shaded region indicating favorability for tear propagation. **a** Contour plot of energy release rates by varying pressure and tear depth for longitudinal tear propagation, when $\alpha _{\text {lim}}$ = $30^\circ$, $H_2$/R = 0.2, and $J_m$ = 1. **b** Contour plot of energy release rates by varying pressure and tear depth for circumferential tear propagation, when $\alpha _{\text {lim}}$ = $30^\circ$, $H_2$/R = 0.2, and $J_m$ = 1

**Fig. 9:**
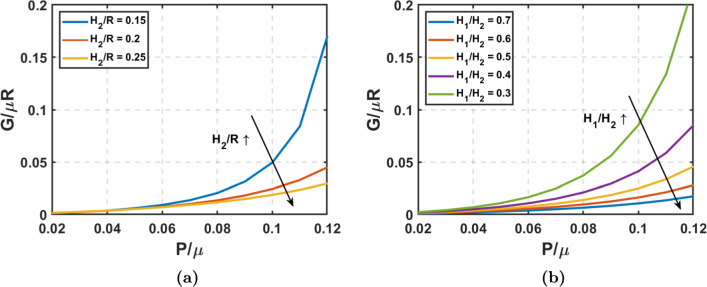
Circumferential tear propagation: Neo-Hookean material: (a) Energy release rate vs. Pressure with varying thickness($H_2$) at $\alpha _{\text {lim}}$ = $30^\circ$ and $H_1$/$H_2$ = 0.5. (b) Energy release rate vs. Pressure with varying tear depth($H_1$/$H_2$) at $\alpha _{\text {lim}}$ = $30^\circ$ and $H_2$/R = 0.2

**Fig. 10:**
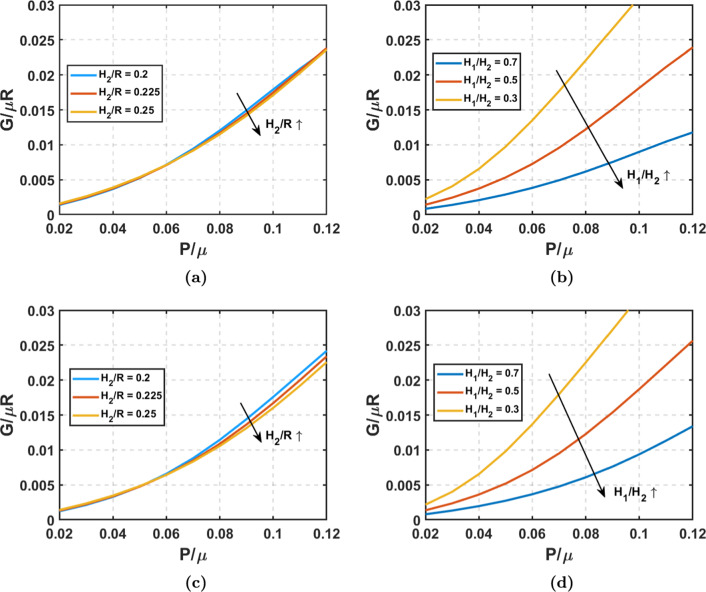
Energy release rate vs. Pressure plots using anisotropic HGO model at $\alpha _{\text {lim}}$ = $30^\circ$, $K_1/\mu$ = 5e-2, and $K_2$ = 10 for longitudinal crack propagation: (a) Varying thickness ($H_2$/R) and $H_1$/$H_2$ = 0.5. (b) Varying tear depth ($H_1$/$H_2$) and $H_2$/R = 0.2. Energy release rate vs. Pressure plots using anisotropic HGO model at $\alpha _{\text {lim}}$ = $30^\circ$, $K_1/\mu$ = 5e-2, and $K_2$ = 10 for circumferential crack propagation: (a) Varying thickness ($H_2$/R) and $H_1$/$H_2$ = 0.5. (b) Varying tear depth($H_1$/$H_2$) and $H_2$/R = 0.2

To have a quantitative insight, we establish a correlation between clinical data on the fracture energy ($\Gamma$) of the abdominal aorta and the energy release rate (*G*) predicted by using Gent hyperelastic model, with $J_m$ = 1 (Horny et al. 2009). Sommer et al. (2008) reported the dissection energy value to be 76 $J/m^2$ in longitudinal and 51 $J/m^2$ in circumferential directions for an effective specimen radius of 20 mm. For the abdominal aorta, the effective shear modulus is approximately 157 kPa (Petterson et al. 2021). Using these material parameters, the computed $\Gamma /\mu R$ is about 0.024 and 0.016 in the longitudinal and circumferential directions, respectively. Based on the results shown in Figs. 4c and 5c, we constructed a safety plot using energy release rate contours by varying tear depth and blood pressure, as depicted in Fig. 7. The analytical model suggests that G exceeds $\Gamma$, indicating favorable conditions for tear propagation in both the longitudinal and circumferential directions under specific geometric configurations (region shaded in gray). When blood pressure is high ($P/\mu \ge$ 0.12), *G* surpasses $\Gamma$ even with small tear depths; however, at lower pressures, the tear depth must be sufficiently large for G to exceed $\Gamma$. Similarly, varying the stiffness parameter ($J_m$) and other geometrical parameters can identify favorable conditions for tear propagation, although the minimal variation of energy release rate with those parameters precludes a detailed discussion of those results. Introducing an anisotropic HGO model could result in slightly different safety profiles than those obtained from an Isotropic Gent material model. However, obtaining representative fit values for $\mu$, $K_1$, and $K_2$ presents a challenge due to their inconsistency in the literature. Nonetheless, we believe this variability would not significantly impact the overall trends observed.

However, the current model has limitations. We simplified the geometry of the aorta, assumed plane strain deformation while neglecting axial stretch, and did not account for the effects of hemodynamics. Our model does not consider the influence of residual stresses. Despite these limitations, our model provides valuable insights into the general patterns of fracture propagation in aortic dissection.

## Conclusion

In this paper, we have developed a fracture mechanics model to investigate the energy release rate of aortic dissection tear propagation in both the longitudinal and circumferential directions. The model incorporates various geometrical and material parameters, accounting for individual differences. Although we have made a membrane assumption for the dissected aortic wall, the results from the model closely align with FEA simulations that do not rely on such assumptions. Furthermore, our findings are in good agreement with clinical observations. We have demonstrated that the energy release rate increases with tear depth and reduction in aortic wall thickness, while it diminishes with aortic stiffening. The impact of the crack length is non-monotonic and is contingent upon the crack propagation direction. Our findings provide valuable insights indicating that individuals with hypertension, i.e., when blood pressure is high, face an increased risk of aortic dissection. Furthermore, even when blood pressure is within the normal range, aortic dissection remains a potential occurrence, contingent upon specific combinations of geometrical and material properties. While our current model is simplistic and entails several assumptions, we can enhance its realism in future works by accounting for the effects of axial stretch.

## Figures and Tables

**Algorithm 1:**
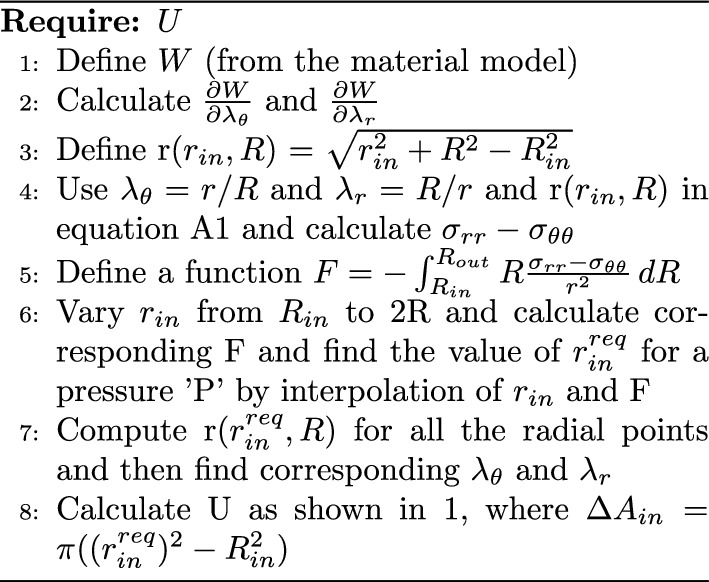
Calculate Potential energy U for normal aorta

**Fig. 8:**
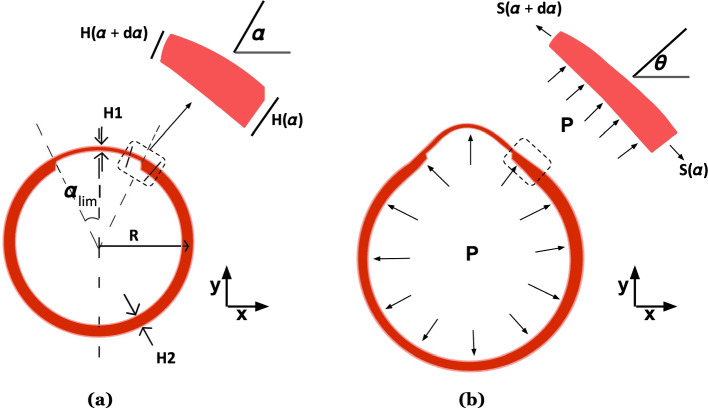
Depiction of aortic membrane used to model the dissected section of aorta: **a** Stress-free (undeformed) state. **b** Deformed state

**Algorithm 2:**
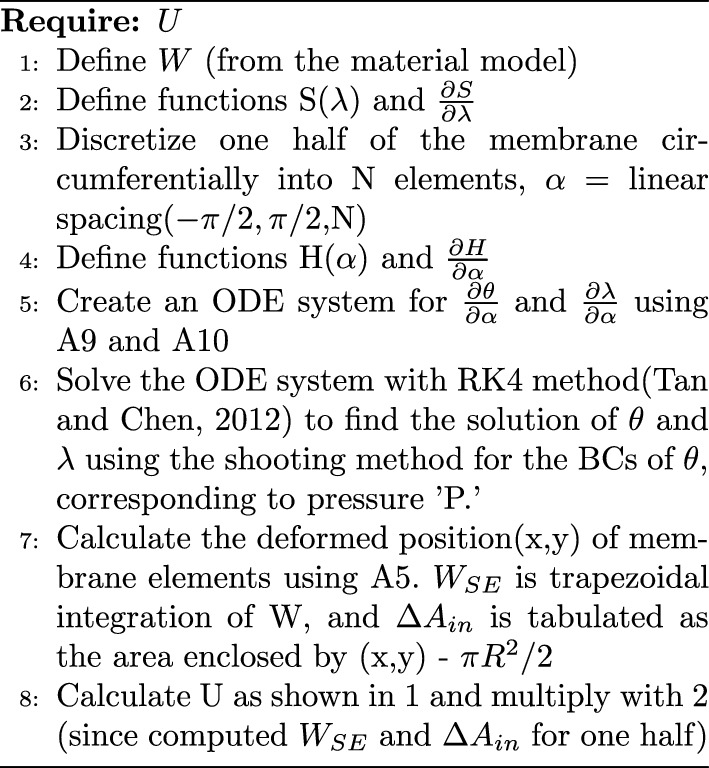
Calculate potential energy U for dissected aorta

## Appendix A Equilibrium

### A.1 Normal aortic section

In the absence of any crack, the section of the normal aorta is axisymmetric, with pressure acting along the inner wall in the radial direction. So, we only have the deformation variation along the thickness. The stretches in the aortic wall are given as:

$$\begin{aligned} \begin{aligned} \lambda _{1} = \lambda _{\theta } = \lambda \text {, } \lambda _{2} = \lambda _{r} = 1/\lambda \text { and } \lambda _{3} = \lambda _{z} = 1 \end{aligned} \end{aligned}$$

We know that $\lambda _{\theta } = r/R$, where r is the deformed material position, and R is the initial material position. We can relate r and R using the incompressibility condition $\pi (r^2 - r_{in}^2) = \pi (R^2 - R_{in}^2)$, where $R_{in}$ and $r_{in}$ are the radii of the inner aortic wall in the undeformed state and after deformation. This helps us define the radial deformation *r* of any material point in terms of a single variable $r_{in}$ and initial radius R. For an incompressible material, the stresses and strain energy density function are related as

A1 $$\begin{aligned} \begin{aligned} \sigma _{rr} - \sigma _{\theta \theta }&= \lambda _r\frac{\partial W}{\partial \lambda _r} - \lambda _{\theta }\frac{\partial W}{\partial \lambda _{\theta }} \end{aligned} \end{aligned}$$

In the axisymmetric case, we have the mechanical equilibrium equation in cylindrical coordinates in the radial direction, given as:

A2 $$\begin{aligned} \frac{\partial \sigma _{rr}}{\partial r}+\frac{\sigma _{rr} - \sigma _{\theta \theta }}{r} = 0. \end{aligned}$$

Integrating over the thickness, we get:

A3 $$\begin{aligned} \begin{aligned} P&= - \int _{R_{\text {in}}}^{R_{\text {out}}} R\frac{\sigma _{rr} - \sigma _{\theta \theta }}{r^2} \,dR \end{aligned} \end{aligned}$$

Using (A1) in (A3) and the corresponding strain energy density function from 2.2, we get an integral equation relating blood pressure ’P’ and $r_{\text {in}}$, which can be solved to get the deformation field along the thickness. A simple algorithm for finding the deformation and potential energy of normal aorta is given in Algorithm 1

### A.2 Dissected aortic section

For the dissected section of the aorta, because of the presence of a crack, the section of the aorta is not axisymmetric but has symmetry along the *y*-axis. For simplicity, we model the aortic wall with crack as a thin single-layer membrane undergoing homogeneous deformation along the thickness of the aortic wall.

Consider a small element of length R$d\alpha$ at an angle ‘$\alpha$’ as shown in Fig. 8a. After deformation, its length increases to dL, and the stretch of the element is given as:

A4 $$\begin{aligned} \begin{aligned} \lambda&=\frac{\text {d} L}{R \text {d} \alpha } \end{aligned} \end{aligned}$$

The deformed position of the element can be related to its stretch as follows:

A5 $$\begin{aligned} \begin{aligned} \frac{\text {d} x}{\text {d} \alpha }&=-R \lambda \sin \theta \\ \frac{\text {d} y}{\text {d} \alpha }&=R \lambda \cos \theta \end{aligned} \end{aligned}$$

The stretches in the element is given as $\lambda _{1} = \lambda$, $\lambda _{2} = 1/\lambda$ , and $\lambda _{3} = \lambda _{z} = 1$, and the membrane stress (*S*) is given by:

A6 $$\begin{aligned} \begin{aligned}&S= \frac{\partial W}{\partial \lambda } \quad \end{aligned} \end{aligned}$$

For the small element after deformation (Fig. 8b), balancing force along the *x*-axis gives:

A7 $$\begin{aligned} \begin{aligned} \quad P \lambda R \cos \theta -\frac{\text {d}}{\text {d} \alpha }(\text {SH} \sin \theta )= 0 \quad \end{aligned} \end{aligned}$$

Balancing force along the *z*-axis gives us:

A8 $$\begin{aligned} \begin{aligned} \quad P \lambda R \sin \theta +\frac{\text {d}}{\text {d} \alpha }\left( \text {SH} \cos \theta \right) =0 \quad \end{aligned} \end{aligned}$$

Solving (A7) and (A8) gives:

A9 $$\begin{aligned}{} & {} \frac{\text {d} \theta }{\text {d} \alpha }=\frac{P \lambda R}{\text {SH}} \quad \end{aligned}$$

A10 $$\begin{aligned}{} & {} \frac{\text {d} \lambda }{\text {d} \alpha }=\frac{-S \frac{\partial H}{\partial \alpha }}{H \frac{\partial S}{\partial \lambda }} \quad \end{aligned}$$

The model has symmetry along the y-axis, thus we use the following boundary conditions:

$$\begin{aligned} \begin{aligned}&\theta = -90^\circ \quad @ \alpha = -90^\circ \\&\theta = 90^\circ \quad @ \alpha = 90^\circ \\&x=0 \quad @ \alpha =90^\circ , 90^\circ \end{aligned} \end{aligned}$$

For a smooth transition of membrane thickness from TLWT ($H_2$) to FLWT ($H_1$) in the false lumen, we assume the thickness function to be as follows:

$$\begin{aligned} \begin{aligned} H(\alpha )= \hspace{19em}\\ {\left\{ \begin{array}{ll}H_2 &{} - 90^\circ<\alpha< \alpha _{\text {lim}} -\frac{1}{2}^\circ \\ H_1 + (H_2-H_1)C &{} \alpha _{\text {lim}} - \frac{1}{2}^\circ \le \alpha \le \alpha _{\text {lim}} + \frac{1}{2}^\circ \\ H_1 &{} \alpha _{\text {lim}} + \frac{1}{2}^\circ<\alpha < 90^\circ \end{array}\right. } \end{aligned} \end{aligned}$$

here, C = $\frac{\left( \alpha - ( \alpha _{\text {lim}} + \frac{1}{2}^\circ ) \right) ^{2}}{ (1^\circ )^{2}}$. By solving the system of ODEs given in A5, A9, and A10 for $\lambda$, $\theta$, x, and y, we get the stretch and deformation field of the membrane. Algorithm 2 describes how to solve the ODEs and calculate U for the dissected aorta.
